# Pancreatic Head Cancer and Intrahepatic Cholangiocarcinoma Occurring After Surgery for Congenital Biliary Dilatation Associated With Pancreaticobiliary Maljunction: A Case Report

**DOI:** 10.7759/cureus.100394

**Published:** 2025-12-30

**Authors:** Satoshi Yokoyama, Kentaro Yasuchika, Ryohei Aoyama, Masaya Tsubakiyama, Yusuke Sakura

**Affiliations:** 1 Surgery, Japanese Red Cross Wakayama Medical Center, Wakayama, JPN

**Keywords:** biliary reconstruction, cholangiocarcinoma, congenital biliary dilatation, pancreatic cancer, pancreaticobiliary maljunction, reoperation

## Abstract

Congenital biliary dilatation (CBD), frequently associated with pancreaticobiliary maljunction (PBM), predisposes patients to biliary tract malignancies due to chronic reflux of pancreatic juice into the biliary epithelium. Although biliary diversion reduces carcinogenic risk, malignancy may still arise many years after surgery. A 69-year-old woman underwent choledochal cyst excision and Roux-en-Y hepaticojejunostomy at age 52. Seventeen years later, she developed pancreatic head adenocarcinoma, treated with subtotal stomach-preserving pancreaticoduodenectomy (SSPPD) while preserving and reusing the original Roux limb. Six months later, she developed intrahepatic cholangiocarcinoma, requiring an extended right hepatectomy with reconstruction using the same preserved Roux limb. She remains disease-free 18 months after surgery. Patients with CBD and PBM remain at lifelong risk of malignancy, especially when surgery is performed in adulthood. Reuse of a preserved Roux limb is a feasible and effective strategy in complex reoperations.

## Introduction

Pancreaticobiliary maljunction (PBM) is a congenital anomaly characterized by an abnormally long common channel located outside the duodenal wall, permitting reciprocal reflux of pancreatic and biliary secretions [1]. This abnormal reflux results in chronic epithelial injury, inflammation, and subsequent carcinogenesis within the pancreatobiliary system [2].

Congenital biliary dilatation (CBD), which frequently coexists with PBM, is therefore associated with a lifelong risk of malignancy [3]. Although excision of the extrahepatic bile duct with biliary diversion is the standard treatment, long-term follow-up studies have demonstrated that malignancies may continue to develop decades after surgery [3-7]. In particular, adult-onset CBD-defined as surgery performed in adulthood-may confer a higher lifetime carcinogenic risk due to prolonged exposure to pancreaticobiliary reflux prior to definitive correction.

The malignancies most commonly reported after CBD excision are extrahepatic cholangiocarcinoma and gallbladder cancer [3-7]. In contrast, pancreatic cancer is relatively rare in patients with PBM [8,9], and intrahepatic cholangiocarcinoma (IHCC) developing long after cyst excision has been reported in only a limited number of cases [4-7,10,11]. Sequential occurrence of pancreatic cancer followed by IHCC in a single patient is therefore exceptionally rare.

Here, we report a unique case of metachronous pancreatic head adenocarcinoma followed by IHCC occurring 17 years after CBD surgery associated with PBM. This case highlights the lifelong carcinogenic risk even decades after surgery and illustrates the feasibility of reusing a preserved Roux-en-Y limb during repeated major hepatopancreatobiliary procedures.

## Case presentation

A 69-year-old woman presented with intermittent upper abdominal pain lasting several weeks. Laboratory evaluation revealed an elevated serum carbohydrate antigen 19-9 (CA19-9) level of 78.8 U/mL, while the carcinoembryonic antigen (CEA) level was within the normal range at 2.7 ng/mL. She had undergone excision of a congenital choledochal cyst with Roux-en-Y hepaticojejunostomy at 52 years of age for CBD associated with PBM. Her baseline performance status was good (ECOG 0), and she had no significant medical comorbidities.

Pancreatic head cancer

**Figure 1:**
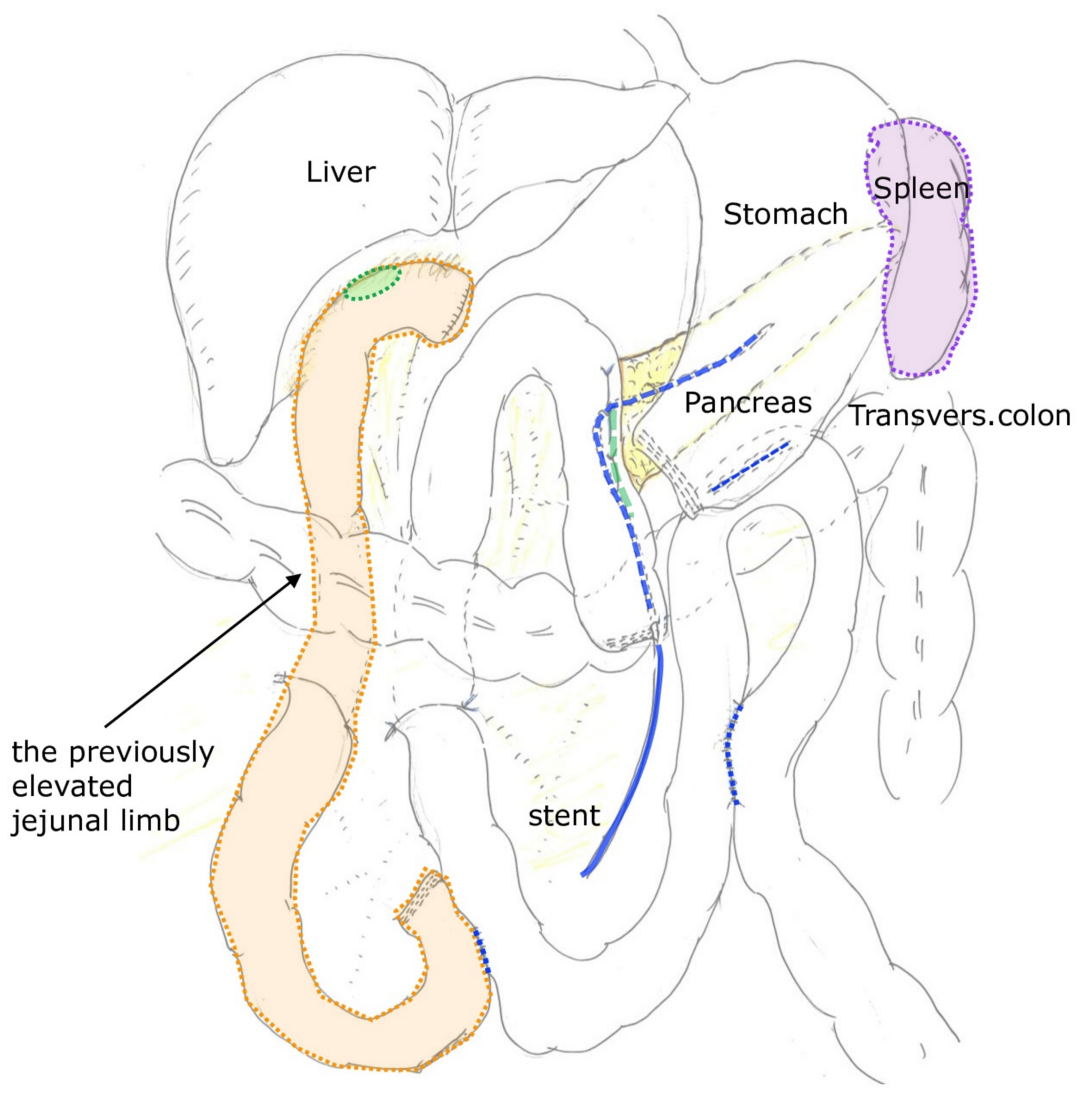
Surgical findings and reconstruction during pancreatic head cancer surgery Intraoperative findings and schematic illustration of subtotal stomach-preserving pancreaticoduodenectomy (SSPPD). The previously constructed Roux-en-Y limb from prior congenital biliary dilatation surgery was carefully preserved. The original hepaticojejunostomy remained intact and was reused for reconstruction, thereby avoiding additional bowel resection or creation of a new biliary-enteric anastomosis (author’s original illustration).

Contrast-enhanced computed tomography (CT) and magnetic resonance cholangiopancreatography (MRCP) revealed a pancreatic head mass without evidence of distant metastasis. Endoscopic ultrasound-guided biopsy confirmed adenocarcinoma. Subtotal stomach-preserving pancreaticoduodenectomy (SSPPD) was performed. The original Roux-en-Y limb and biliary-enteric anastomosis were carefully preserved and reused for reconstruction (Figure 1).

The postoperative course was uneventful, and serum tumor marker levels normalized.

Histopathological examination demonstrated moderately differentiated tubular adenocarcinoma with negative surgical margins (R0) and no lymph node metastasis (pT2N0M0). Based on pathological findings and patient preference, no adjuvant chemotherapy was administered.

Intrahepatic cholangiocarcinoma

**Figure 2:**
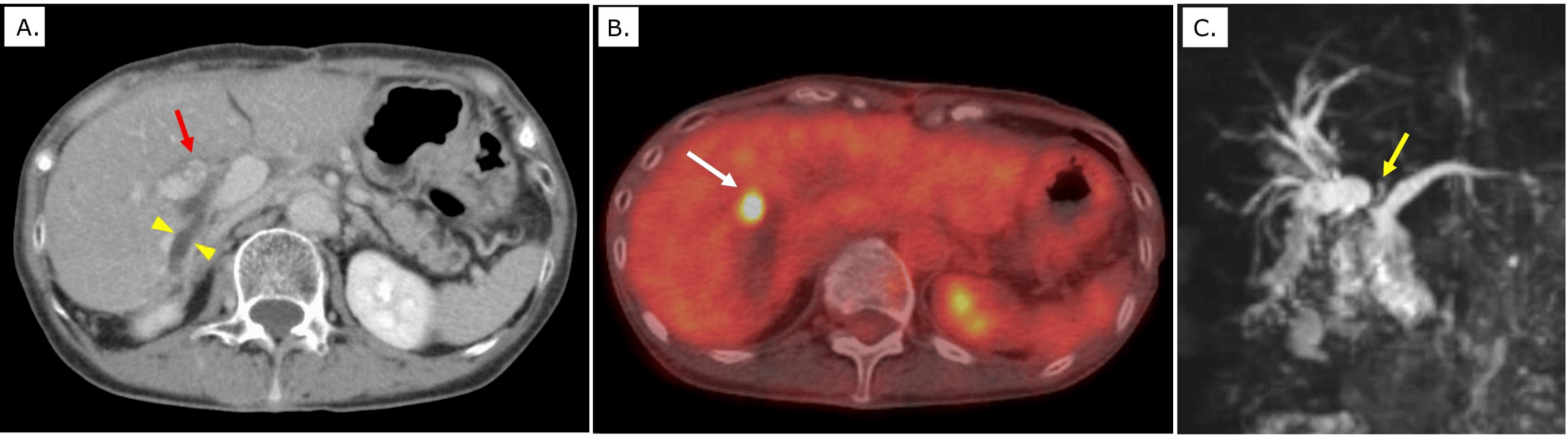
Imaging findings and preoperative simulation for intrahepatic cholangiocarcinoma A: Contrast-enhanced computed tomography (CT) demonstrates dilation of the right intrahepatic bile duct (yellow arrowhead) and a soft-tissue density adjacent to the jejunal anastomosis (red arrow). B: Positron emission tomography–CT (PET-CT) reveals nodular fluorodeoxyglucose (FDG) uptake within the soft-tissue lesion near the anastomosis (white arrow), suggestive of malignancy. C: Magnetic resonance cholangiopancreatography (MRCP) shows dilation of the right intrahepatic bile ducts with a filling defect between the bile ducts and the anastomosed jejunal limb (yellow arrow).

Six months later, during routine postoperative surveillance, serum tumor markers were again elevated, with a CEA level of 5.6 ng/mL and a CA19-9 level of 518.0 U/mL. Contrast-enhanced CT, Positron emission tomography-CT (PET-CT) and magnetic resonance imaging revealed a hepatic hilar mass (Figure 2A-2C). Percutaneous biopsy confirmed IHCC.

**Figure 3:**
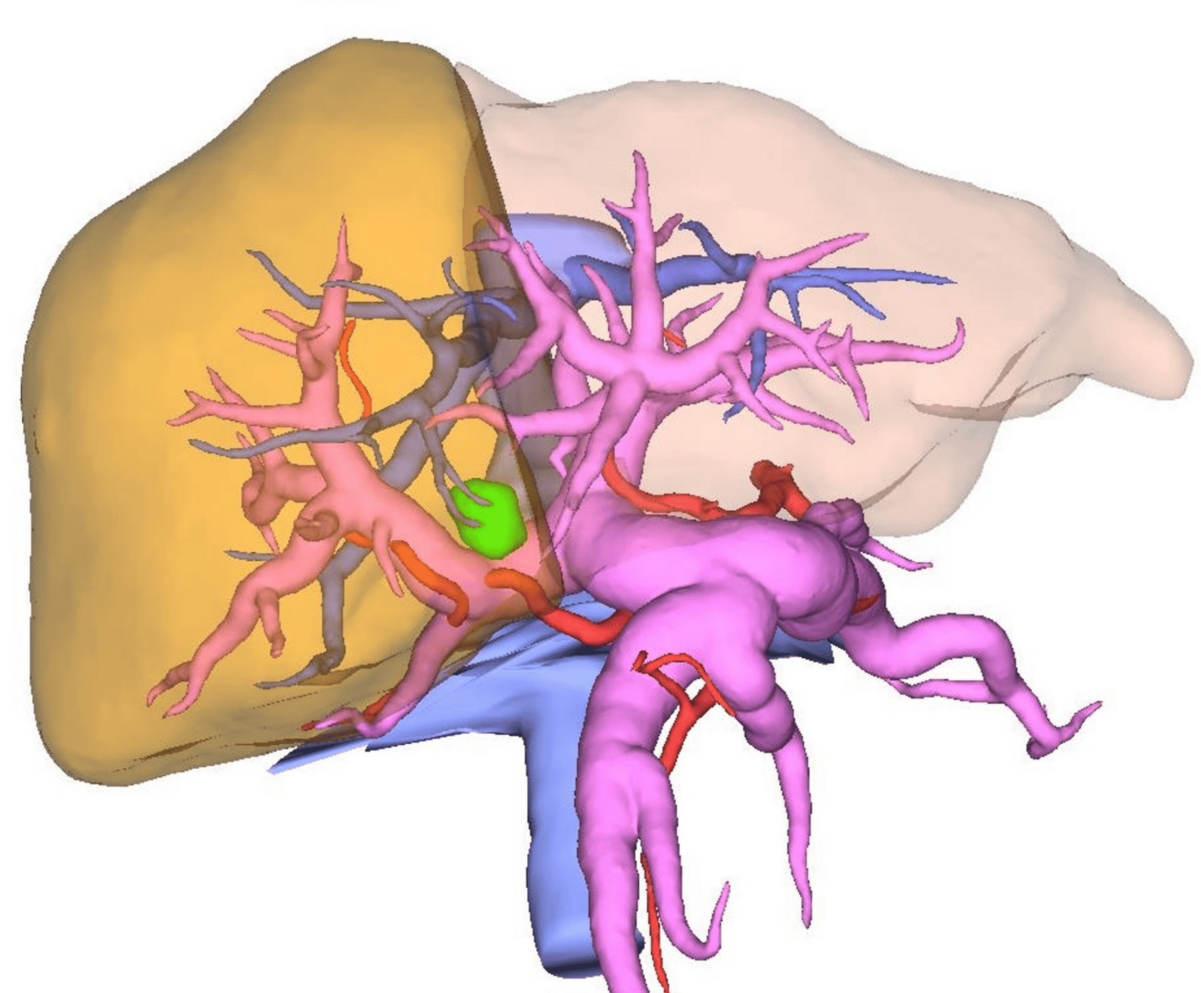
Preoperative simulation images Preoperative three-dimensional simulation using SYNAPCE VINCENT® visualizing the spatial relationship between the tumor, and adjacent vascular structures. This simulation facilitated precise assessment of tumor extent, determination of the hepatic transection line, and formulation of an optimal operative strategy.

Preoperative three-dimensional simulation using SYNAPCE VINCENT® was performed to visualize the vascular and biliary anatomy and to support operative planning (Figure 3). This simulation enabled a precise assessment of tumor location, its relationship to adjacent structures, and the anticipated resection line.

**Figure 4:**
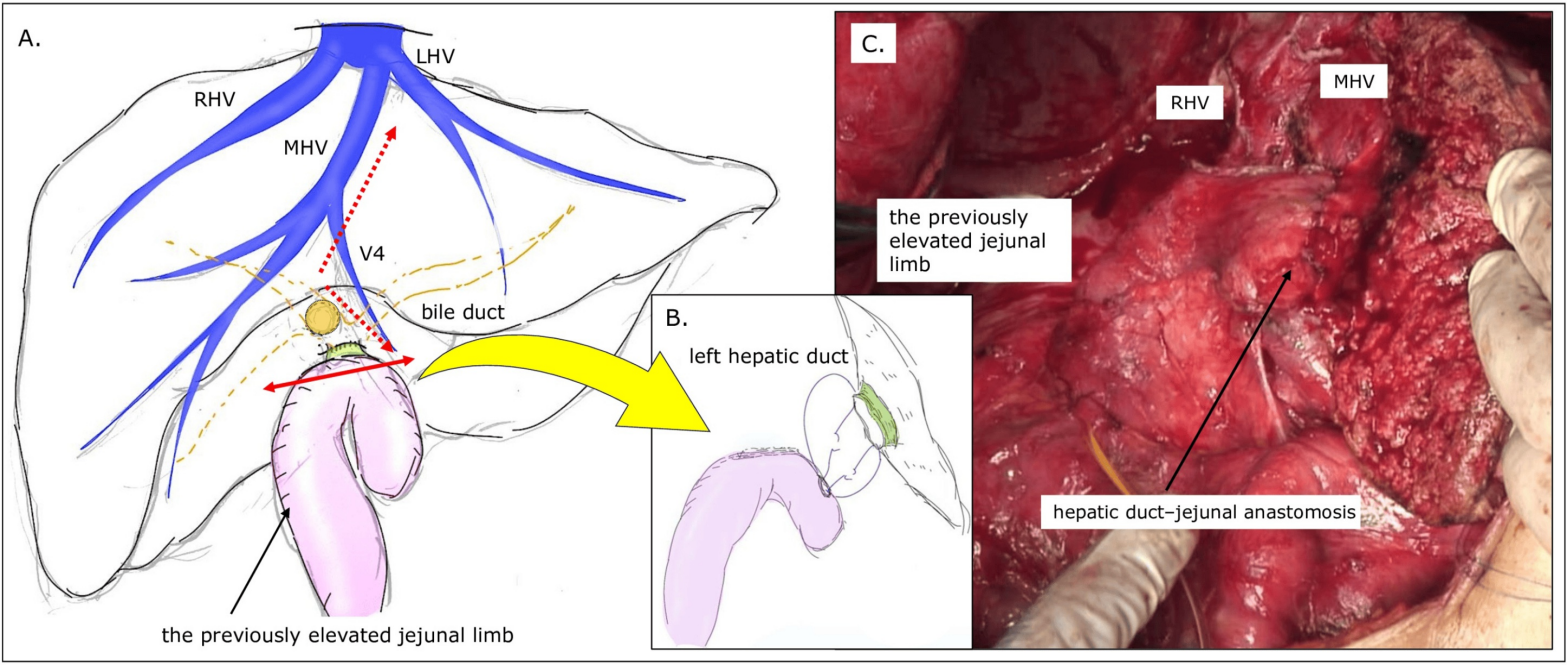
Surgical schema and intraoperative findings during hepatectomy A: Author’s original surgical schema illustrating transection of the elevated jejunal limb just below the hepaticojejunostomy created during the initial surgery (red double-headed arrow). The planned hepatic transection line is indicated by the red dashed arrow. B: Schematic illustration showing reuse of the preserved Roux-en-Y limb for reconstruction via left hepatic duct–jejunal anastomosis. C: Intraoperative photograph demonstrating an extended right hepatectomy with en bloc resection of the previous hepaticojejunostomy. The preserved Roux limb was subsequently reused to construct a left hepatic duct–jejunal anastomosis. MHV: Middle hepatic vein; LHV: Left hepatic vein; RHV: Right hepatic vein

The patient subsequently underwent an extended right hepatectomy with en bloc resection of the previous hepaticojejunostomy. The preserved Roux-en-Y limb was reused for left hepatic duct-jejunal anastomosis (Figure 4A-4C). The postoperative course was uneventful, and no adjuvant chemotherapy or radiotherapy was administered. She remains alive without evidence of recurrence 18 months after the second surgery and continues regular surveillance with cross-sectional imaging and tumor marker assessment.

Pathology

**Figure 5:**
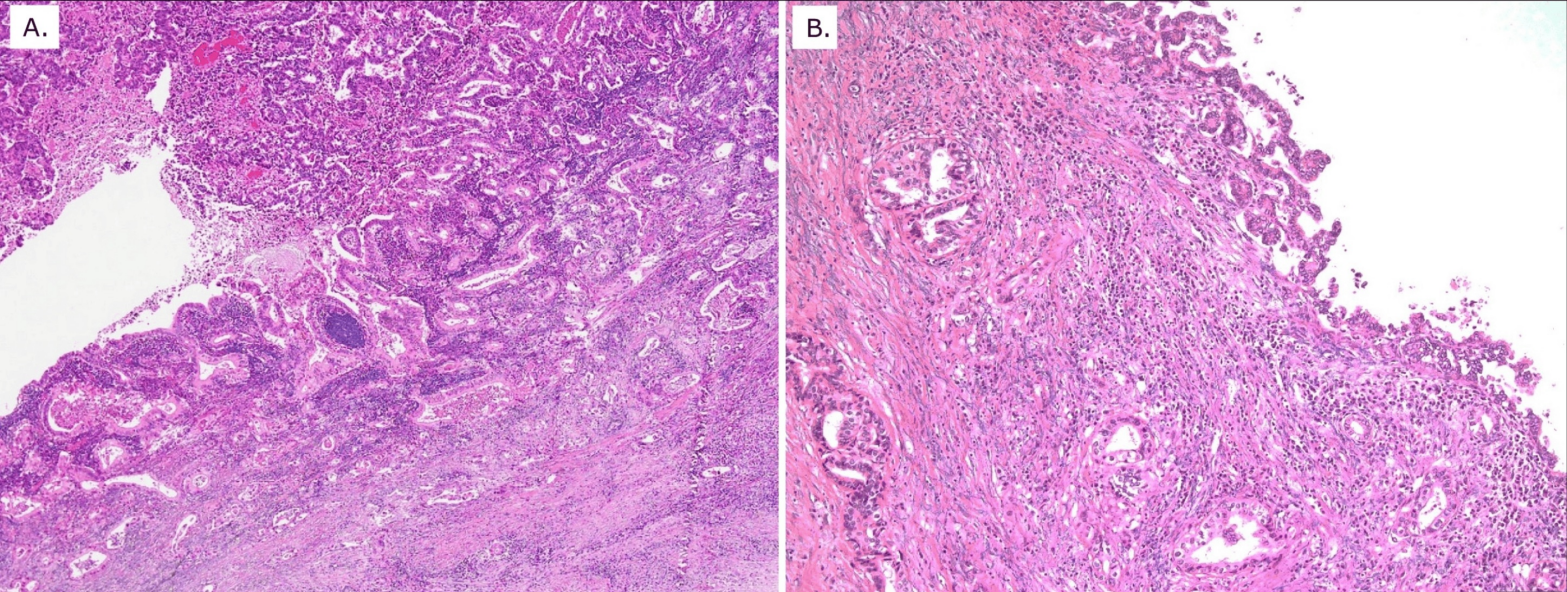
Histopathological findings of the two primary malignancies A: Histopathological examination of the pancreatic lesion revealed moderately differentiated tubular adenocarcinoma confined to the pancreatic head, without involvement of the extra-pancreatic bile duct. Adenocarcinoma in situ (AIS) was observed extending from the ampullary epithelium into the common channel and main pancreatic duct, consistent with pancreatic head adenocarcinoma. B: Histopathological examination of the hepatic lesion demonstrated AIS arising from the intrahepatic bile duct epithelium with associated stromal invasion, consistent with intrahepatic cholangiocarcinoma. No histologic continuity between the pancreatic and hepatic tumors was identified, supporting the diagnosis of metachronous double primary cancers.

Histopathological examination of the pancreatic lesion demonstrated moderately differentiated tubular adenocarcinoma (Figure 5A). The hepatic tumor was diagnosed as moderately differentiated IHCC arising from the right intrahepatic bile duct (Figure 5B). 

There was no histologic continuity between the two tumors, confirming the diagnosis of metachronous double primary cancers rather than recurrence or metastatic disease.

## Discussion

PBM results in continuous exposure of the biliary tract to pancreatic enzymes and bile acids, leading to chronic epithelial injury, inflammation, and carcinogenesis [1,2]. Although cyst excision significantly reduces this risk, long-term studies have shown that the cumulative incidence of malignancy continues to increase, particularly beyond 15-30 years after surgery [3-7,10,11]. Adult-onset CBD may be associated with an even higher lifetime risk due to prolonged preoperative exposure to pancreaticobiliary reflux.

In the present case, CBD excision was performed at 52 years of age, suggesting that carcinogenic processes may have already been initiated. This prolonged exposure may explain the subsequent development of two distinct primary malignancies-pancreatic head adenocarcinoma followed by IHCC. While pancreatic cancer after CBD excision is rare [8,9], IHCC has been reported in several long-term follow-up studies [4-7,10,11]. We reviewed the literature, and previous cases of malignancy after cyst excision are summarized in Table 1. To our knowledge, this is the first English-language report describing sequential pancreatic head adenocarcinoma followed by IHCC after CBD excision associated with PBM.

**Table 1:** Reported cases of pancreatic and biliary malignancies after choledochal cyst excision or in association with pancreaticobiliary maljunction This table summarizes representative English-language case reports and selected series documenting pancreatic and/or biliary malignancies arising after choledochal cyst excision or in patients with pancreaticobiliary maljunction (PBM). The table is not intended as a systematic review; some cohort and review articles report additional cases not individually listed. References were selected based on clear documentation of latency interval, tumor type, and surgical history to illustrate long-term carcinogenic risk after cyst excision. PDAC: Pancreatic invasive ductal adenocarcinoma; IHCC: Intrahepatic cholangiocarcinoma; CC: Cholangiocarcinoma; CBD: Common bile duct; Hilar CC: hilar cholangiocarcinoma

No.	Citation	Patient (age/sex)	Age at initial surgery	Interval to malignancy	Tumor site / histology	Key notes
1	Eriguchi N et al., 2001 [9]	42, F	25 years	17 years	PDAC	Pancreatic carcinoma after choledochal cyst excision.
2	Ohashi T et al., 2013 [3]	Series	Various	13–32 years	Biliary malignancies	Large series with long intervals.
3	Nishiyama R et al., 2011 [4]	61, F	28 years	33 years	IHCC	IHCC with chronic inflammation.
4	Shimamura K et al., 2009 [10]	44, M	10 years	34 years	IHCC	Giant hepatic tumor decades later.
5	Kumamoto T et al., 2014 [5]	40, F	12 years	28 years	IHCC	Long-term carcinogenesis.
6	Kato H et al., 2015 [6]	46, F	14 years	32 years	CC	Arising from remnant intrapancreatic duct.
7	Chen Y et al., 2016 [7]	59, F	46 years	13 years	Hilar CC	Metachronous hilar CC.
8	Park SW et al., 2012 [11]	42, F	33 years	9 years	CBD cancer	Cancer from remnant intrapancreatic duct.
9	Present case	69, F	52 years	17 years + 6 months	PDAC & IHCC	First case of both cancers after excision.

The occurrence of metachronous tumors at different anatomical sites can be explained by a field carcinogenesis phenomenon. Chronic pancreaticobiliary reflux creates a diffuse carcinogenic environment throughout the pancreatobiliary epithelium, allowing independent tumors to arise over time without histologic continuity.

Despite a shared carcinogenic background, pancreatic ductal adenocarcinoma and IHCC likely arise through different biological pathways. Pancreatic cancer is thought to develop via pancreatic intraepithelial neoplasia driven by chronic inflammation and genetic instability, whereas IHCC is associated with prolonged biliary epithelial injury, cholestasis, and inflammatory changes within the intrahepatic bile ducts.

This case also underscores the importance of lifelong surveillance, even decades after cyst excision. Although evidence-based surveillance protocols are lacking, long-term follow-up incorporating periodic imaging and tumor marker assessment may be reasonable. The delayed emergence of malignancy in this patient highlights the persistence of carcinogenic risk despite definitive surgery.

From a surgical standpoint, this case offers important educational value for reoperative planning. Reuse of a preserved Roux-en-Y limb during repeated hepatopancreatobiliary operations was safe and effective, avoided additional bowel resection, and simplified reconstruction in a complex operative field.

The limitations of this report include its single-case design and the absence of genetic or molecular analyses, which preclude causal inference or generalization. Nevertheless, the long latency, clear pathological distinction between tumors, and successful surgical management provide valuable insight into long-term oncologic risk and operative strategy in patients with PBM after CBD excision.

## Conclusions

CBD associated with PBM carries a lifelong risk of malignancy, even decades after cyst excision, particularly when surgery is performed in adulthood. Sequential development of pancreatic head adenocarcinoma and intrahepatic cholangiocarcinoma, as demonstrated in this case, underscores the need for sustained vigilance and lifelong surveillance. In addition, the reuse of a preserved Roux-en-Y limb represents a feasible and safe reconstructive option in complex hepatopancreatobiliary reoperations.
